# Medullary Thyroid Cancer: Molecular Drivers and Immune Cellular Milieu of the Tumour Microenvironment—Implications for Systemic Treatment

**DOI:** 10.3390/cancers16132296

**Published:** 2024-06-22

**Authors:** Alexander J. Papachristos, Hazel Serrao-Brown, Anthony J. Gill, Roderick Clifton-Bligh, Stanley B. Sidhu

**Affiliations:** 1 Northern Clinical School, Sydney Medical School, Faculty of Medicine and Health, University of Sydney, Sydney, NSW 2006, Australia; alex.papachristos@sydney.edu.au (A.J.P.);; 2 Endocrine Surgical Unit, Royal North Shore Hospital, Sydney, NSW 2065, Australia; 3 NSW Health Pathology, Department of Anatomical Pathology, Royal North Shore Hospital, Sydney, NSW 2065, Australia; 4 Cancer Diagnosis and Pathology Group, Kolling Institute of Medical Research, Royal North Shore Hospital, Sydney, NSW 2065, Australia; 5 Department of Endocrinology, Royal North Shore Hospital, Sydney, NSW 2065, Australia

**Keywords:** medullary thyroid cancer, tumour microenvironment, targeted therapy

## Abstract

**Simple Summary:**

Medullary thyroid carcinoma (MTC) is driven by a small number of pathogenic genetic variants and tumours usually exhibit a correspondingly low tumour mutational burden. This reduces tumour visibility to the immune system and impacts the immune cell profile of the tumour microenvironment. In the last decade targeted pathway inhibitors have revolutionized the therapeutic landscape for patients with advanced disease, with increasing options for systemic therapy tailored to the molecular signature of the tumour. Therefore, understanding the molecular basis of disease, pathogenesis of immune evasion and mechanisms of escape of pathway inhibition is of paramount importance. Here, we summarize genetic and molecular drivers of MTC and their relevance to tumour immunogenicity, the cellular milieu of the tumour microenvironment, and response to targeted therapy.

**Abstract:**

In this review, we explore the underlying molecular biology of medullary thyroid carcinoma (MTC) and its interplay with the host immune system. MTC is consistently driven by a small number of specific pathogenic variants, beyond which few additional genetic events are required for tumorigenesis. This explains the exceedingly low tumour mutational burden seen in most MTC, in contrast to other cancers. However, because of the low tumour mutational burden (TMB), there is a correspondingly low level of tumour-associated neoantigens that are presented to the host immune system. This reduces tumour visibility and vigour of the anti-tumour immune response and suggests the efficacy of immunotherapy in MTC is likely to be poor, acknowledging this inference is largely based on the extrapolation of data from other tumour types. The dominance of specific *RET* (REarranged during Transfection) pathogenic variants in MTC tumorigenesis rationalizes the observed efficacy of the targeted RET-specific tyrosine kinase inhibitors (TKIs) in comparison to multi-kinase inhibitors (MKIs). Therapeutic durability of pathway inhibitors is an ongoing research focus. It may be limited by the selection pressure TKI treatment creates, promoting survival of resistant tumour cell clones that can escape pathway inhibition through binding-site mutations, activation of alternate pathways, and modulation of the cellular and cytokine milieu of the tumour microenvironment (TME).

## 1. Introduction

Medullary thyroid carcinoma (MTC) is a rare neuroendocrine malignancy that arises from the parafollicular ‘C’ cells of the thyroid gland and accounts for 1–2% of all thyroid cancers [1]. It carries a poorer prognosis than differentiated thyroid cancer; however, there is significant variability in its clinical course; some patients living for decades with low volume nodal disease, whereas others die quickly from rapidly progressive metastases [2,3]. In the last decade, targeted pathway inhibitors have revolutionised the therapeutic landscape for patients with advanced disease, with increasing options for systemic therapy tailored to the molecular signature of the tumour. Therefore, understanding the molecular basis of disease, pathogenesis of immune evasion and mechanisms of escape of pathway inhibition is of paramount importance. In this review, we summarise genetic and molecular drivers of MTC and their relevance to tumour immunogenicity, the cellular milieu of the tumour microenvironment, and response to targeted therapy.

## 2. Materials and Methods

A comprehensive literature review utilising the PubMed database and Google Scholar search engine was performed. Keywords included “medullary thyroid cancer”, “medullary thyroid carcinoma”, “RET”, “tumour microenvironment”, “tyrosine kinase inhibitor”, “tyrosine kinase inhibitor resistance”, “tumour infiltrating lymphocytes” “immunotherapy”, and “targeted therapy”. The search was limited to articles published in English, and primarily focussed on articles published in the last 10 years, with the inclusion of selected relevant papers published outside of this timeframe. Following identification, papers were evaluated to ensure their relevance, appropriate methodology and clinical significance.

## 3. Genetic and Molecular Drivers of Disease

MTC can be broadly classified into hereditary and sporadic disease, with distinct associated molecular pathogenesis. Approximately 25% of MTC cases are ‘hereditary’ and associated with pathogenic germline variants in *RET* (REarranged during Transfection) protooncogene which cause MEN2 syndrome [4], including 5–7% of patients with apparently ‘sporadic’ disease in whom the mutation arises de novo [5]. Patients with pathogenic germline variants in *RET* have a lifetime risk of developing MTC that approaches 100% [6] and inheritance is autosomal dominant. The spectrum of clinical phenotypes includes Multiple Endocrine Neoplasia (MEN) type 2A and type 2B. Familial Medullary Thyroid Cancer (FMTC) syndrome is now recognized as a forme fruste of MEN2A [1]. Within these groups, there is both inter- and intra-family variation in clinical manifestations. Different *RET* mutations confer different levels of risk with respect to the age at which MTC typically develops and associated prognosis. For example, patients with the *RET* codon M918T variant are at the highest risk of aggressive disease and are recommended to undergo thyroidectomy within the first six months of life, whereas patients with *RET* codon C634 variants (responsible for approximately 75% of MEN2A) are at intermediate risk and should undergo thyroidectomy by the age of 5 y [1].

## 4. The *RET* Protooncogene and Hereditary Disease

**Figure 1:**
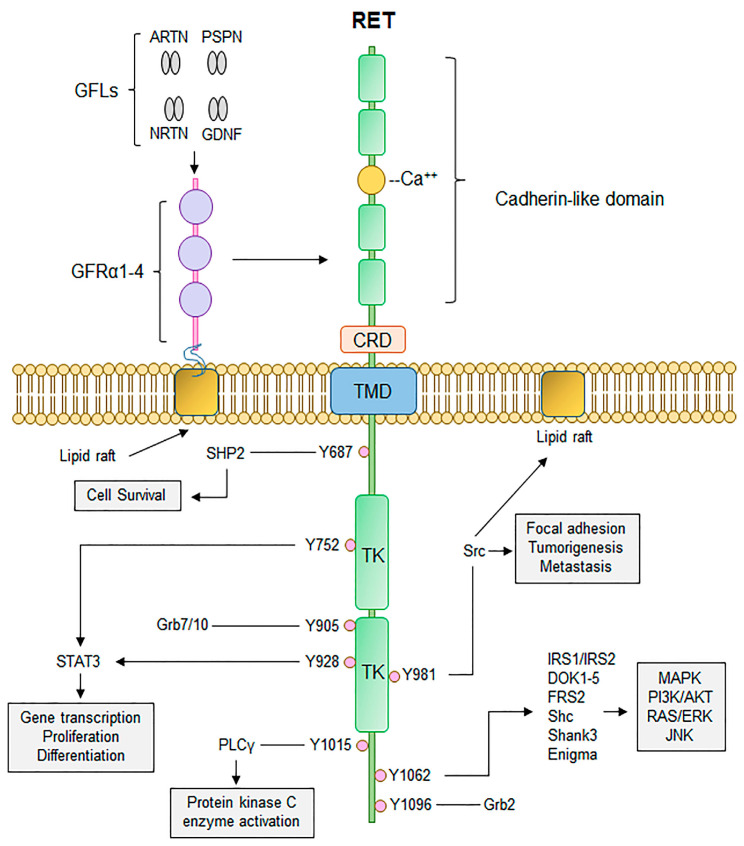
Canonical RET signalling. RET activation occurs upon fulfilment of multiple steps. Binding of GDNF-family ligands (GFLs), to co-receptor GFRα1-4, concurrently with binding of calcium ions to the calcium binding domain, induces recruitment of RET, forming RET-GFRα complex. Formation of RET-GFRα complex brings two RET monomers in close proximity to induce homodimerization and cross phosphorylation of key RET tyrosine residues that recruit adaptor proteins important for propagation of RET signalling, such as PI3K/AKT, MAPK, and RAS/RAF/ERK. Thus, activation of RET signalling ultimately promotes cell proliferation, growth, and survival through activation of multiple downstream signalling cascades. CRD, cysteine-rich domain; TMD, transmembrane domain; TK, tyrosine kinase domain. Adapted from [12]. Published under a Creative Commons Attribution (CC BY) License.

*RET* encodes a transmembrane tyrosine kinase receptor (TKR), composed of an extracellular domain with a cysteine-rich region and four cadherin-like regions, a transmembrane domain, and an intracellular tyrosine kinase domain (Figure 1) [7]. Initially described by Takahashi et al. in the 1980s, it is located on chromosome 10q11.2 [4,8] and plays a crucial role in development of the genitourinary tract and nervous system [9,10,11] as a functional receptor for glial cell-derived neurotrophic factors (GDNFs).

In absence of ligand binding, the RET TKR exists as a single unphosphorylated TKR. With ligand binding, it undergoes dimerization and phosphorylation to activate several intracellular signalling pathways, including the p38 mitogen-activated protein kinase (MAPK) pathway, the phosphatidyl-inositol 3-kinase (PI3K) pathway and the extracellular signal-related kinase (ERK) pathway, which result in cellular proliferation [13]. Pathogenic variants cause constitutional activation and therefore drive downstream pathways independent of ligand binding to the extracellular domain.

The mechanism of oncogenic activation differs depending on the region of the RET TKR that is abnormal. Variants in the extracellular domain trigger ligand-independent dimerization and autophosphorylation, which in turn activate downstream intracellular signalling pathways. In comparison, if the variant occurs in the intracellular tyrosine kinase domain, autophosphorylation occurs without dimerization, which may drive activation of additional downstream pathways that are not typically controlled by the RET TKR when activated through the extracellular domain [14]. In RET fusion events, the transmembrane domain is absent and the kinase domain is constituently active [12].

Several distinct activating *RET* single nucleotide variants, with corollary phenotypic patterns [1], drive the majority of MTC cases; although, *RET* appears to be the only germline driver gene mutation recurrently involved in MTC [15]. The most common is the M918T variant, followed by variants in cysteine codons (C634, C620, C618, C630, C609 and C611). However, with improved recognition and genetic screening technology, over 100 mutation hotspots have been identified [1]. The type of *RET* variant may help to differentiate sporadic and hereditary MTC. For example, C630 variants suggest sporadic disease, whereas mutational hotspots of C609, C611, D631 and V804 suggest hereditary disease [16]. Furthermore, routine genetic screening of MTC patients with apparent sporadic disease results in reclassification of approximately 5–7% of cases as hereditary, mostly within the subgroup of MEN2A [17].

The specific driver event also informs assessment of disease biology and associated phenotypic manifestations [1], through activation of distinct intracellular signalling cascades with associated transcriptional implications and gene expression [18]. For example, neuronatin (NNAT), cell division cycle 14B (CDC14B) and protein tyrosine phosphatase receptor type T (PTPRT) are upregulated in patients with variants in the cysteine-rich region of the RET TKR extracellular domain (“MEN2A-like”) [14], whereas gamma-aminobutyric acid type A receptor subunit rho1 (GABRR1) and neurotrophic tyrosine receptor kinase 3 (NTRK3) are upregulated up to five-fold in patients with variants in the intracellular tyrosine kinase domain (“MEN2B-like”), which is associated with an aggressive biological phenotype [19]. Variants associated with MEN2B have also been demonstrated to suppress expression of genes required for recruitment and action of NK cells and T-cells in the tumour microenvironment (TME), such as chemokine C-X3-C motif ligand 1 (*CX3CL1*), with inflammatory cell infiltrates only seen in MEN2A/FMTC-associated tumours [20]. Interestingly, gene expression profiles of MTC driven by germline (hereditary) compared to somatic (sporadic) variants are not significantly different [14], suggesting activation of similar signalling pathways for a given *RET* mutation [21].

From an oncogenesis perspective, secondary genetic events are required before MTC develops in patients with pathogenic germline *RET* variants [22], analogous to the ‘two-hit’ hypothesis [23]. However, the second event (e.g., loss of the normal allele or duplication of the RET-mutant allele [24]) may require only a minor genetic alteration, particularly in association with an M918T mutation, as overall mutational burden in MTC is exceedingly low [15], and oncogenic progression of C cells from normal, to hyperplastic, to malignant evolves rapidly, and may occur in the first months of life [25].

## 5. Sporadic Disease

Although MTC is associated with a low mutational burden [15], most tumours are driven by a specific identifiable pathogenic driver variant [26]. In sporadic disease, somatic pathogenic *RET* variants drive approximately half of cases, with rat sarcoma virus (*RAS*) mutations driving 70% of RET wild-type tumours [27]. Interestingly, these two dominant driver mutations appear to be mutually exclusive. In addition to *RET* and *RAS*, uncommon variants in BRAF and NF1 have also been described as oncogenic drivers and there is an increased risk of MTC in NF1 syndrome [28]. There remains a small proportion of sporadic MTC cases with unknown driver events.

Additional pathogenic variants in tumour suppressor genes or DNA repair genes, as well as upregulation of genes that have a synergistic effect in oncogenesis may also contribute to disease progression, with mutations in tumour protein 53 (*TP53*), cluster of differentiation 117 (*KIT*), mutS homologue 6 (*MSH6*), mutL homologue 1 (*MLH1*), ataxia-telangiectasia mutated (*ATM*), von Hippel-Lindau (*VHL*), phosphatase and tensin homolog (*PTEN*), cyclin dependent kinase 2A (*CDKN2A*) and serine/threonine kinase 11 (*STK11*) seen in *RET* or *RAS* mutant tumours [16,29]. However, these are secondary genetic events that contribute to a more aggressive tumour phenotype rather than primary driver variants [30]. Similarly, epigenetic factors including microRNA overexpression (miR-183 and miR-375) may modulate the clinical phenotype of disease [31].

## 6. Molecular Subtyping

More recently, a proteome-based stratification of MTC into three molecular subtypes (metabolic, basal and mesenchymal) has been proposed, with distinct genetic and epigenetic profiles [28]. For example, tumours driven by the RET M918T variant were predominantly of the mesenchymal subtype, with prevalent upregulation of extracellular matrix pathways, frequent epigenetic DNA methylation and the poorest prognosis, whereas RAS-driven tumours were more likely to be of the metabolic subtype, with upregulation of pathways related to cellular metabolism, higher frequency of somatic copy number alterations (*CHEK2*, *MUTYH*, *TP53*, *ATM*, *MLH1*), strong activation of the MAPK and PI3K pathways, and associated with an intermediate prognosis [28]. In comparison, the basal subtype was the most genetically stable and carried the best prognosis. It retained a greater degree of neuroendocrine differentiation, with upregulation neuroendocrine markers including CEA, chromogranin A, synaptophysin and neural cell adhesion molecule 1 (NCAM1, CD56), more closely resembling normal C cells. Further studies are required to determine whether this classification system will prove clinically useful.

## 7. Interplay between the Immune System and Medullary Thyroid Cancer

### An Overview of the Immune System and Carcinogenesis

The immune system plays an important role in suppressing cancer development [32]. It has been more than half a century since Burnet described the concept of immunological surveillance as fundamental to the maintenance of tissue homeostasis, occurring through lymphocyte-mediated recognition and elimination of genetically mutated somatic cells [33]. Despite initial scepticism, this hypothesis was supported by observing oncogenic viruses caused tumours with increased frequency in immunocompromised patients. Subsequently, observational studies from around the world have reported immunocompromised patients have higher standardised incidence ratios for the development several cancers with no known viral trigger, including colonic, lung, pancreatic, urothelial, endocrine and malignant melanoma [34].

The immune system can be described as a collection of physiological processes that enable recognition and elimination of foreign, or “non-self” antigens, and is broadly subdivided into innate and adaptive processes. Innate immunity involves recognition of the structural components of an ‘intruder’, known as pathogen-associated molecular patterns, through several germline-encoded pathogen recognition receptors [35]. Cellular mediators of the innate immune response include macrophages, neutrophils, and natural killer cells. Activation leads to antigen presentation, phagocytosis, and apoptosis, as well as expression of pro-inflammatory cytokines. These cytokines trigger an iterative augmentation of the innate immune response, as well as engagement of adaptive immune pathways. Adaptive immunity is mediated by clonal expansion of effector T and B cells, targeted to a specific antigen, initiated by innate immune signals [35].

Most tumour cells express antigens that can be recognised by the immune system. Despite this, cancer cells can adapt to evade detection through a process known as immunoediting [36]. Normally, the immune system is able to clear tumour, or hold it in a state of ‘equilibrium’ through innate (natural killer and dendritic cells) and adaptive (CD4 and CD8 T cells) mechanisms, until mutating tumour cells acquire the ability to evade detection or elimination [37] and therefore progress to a clinically significant pathology [38]. By manipulating the cytokine and chemokine milieu of the TME, cancer cells may suppress activation or efficacy of immune cells, resulting in ‘tolerance’ rather than clearance [7]. This escape from immune surveillance may be considered the ‘seventh hallmark’ of cancer [39], and similarly, the vigour of the immune response to genetically altered tissue has been demonstrated to be a prognostic factor for survival in melanoma [40], colorectal, breast and ovarian cancer [38]. Most published data describing the immune microenvironment in thyroid cancer pertain to differentiated thyroid cancer (papillary thyroid carcinoma and follicular thyroid carcinoma) and anaplastic thyroid cancer [41], and given fundamental differences in the underlying neuroendocrine biology of MTC, the degree to which these results can be extrapolated is uncertain.

## 8. Mechanisms of Immune Evasion

### 8.1. Immune Suppression Mediated by the Tumour Microenvironment

The TME is composed of extracellular matrix, lymphatics, mesenchymal and immune cells, and plays an important role in the suppression of the anti-tumour immune response [4]. The TME differs from physiological tissue, with relative tissue hypoxia, increased acidity due to lactate-producing metabolic processes, and increased reactive oxygen species [42]. Substrates for cellular metabolism are consumed by tumour cells beyond the ability of homeostatic regulation, and secondary by-products accumulate [43]. Infiltrating immune cells require nutrients and a physiological interstitial milieu to mount an effective immune response [44], and hence, the hostile nature of the TME impairs the immune cell effector function. Cancer cells may adapt to rely on aerobic glycolysis instead of oxidation phosphorylation and therefore deplete glucose in the TME required for T cell activation and effector function [45]. Similarly, elevated lactate levels inhibit T cell signalling [46] and reduce production of effector cytokines, including perforin and granzyme B [47].

The cytokine and chemokine milieu of the TME affects the recruitment and differentiation of key immune effector cells and may create either a tumour-inhibiting or tumour-promoting environment. For example, increased expression of immune suppressive cytokines, such as transforming growth factor β (TGF-β), vascular endothelial growth factor (VEGF) and interleukin 10 (IL10) may inhibit anti-tumour immune response [48]. In MTC, the specific *RET* driver variant may alter expression of genes encoding cytokines and chemokines involved with recruitment and stimulation of T cells and NK cells to the TME [20]. Furthermore, recruitment of immune suppressive cells, such as myeloid derived suppressor cells and M2 macrophages, may render effector T cells in the TME dysfunctional through production of immunosuppressive cytokines [49]. Pozdeyev et al. described the effect of myeloid infiltrate in MTC, in which CD163+ M2 macrophages were frequently present [30], producing cytokines and chemokines that promote angiogenesis, including vascular endothelial growth factor (VEGF) and prostaglandin E2 [50]. These macrophages also manifested a dysfunctional phenotype, lacking the Nuclear factor kappa-light-chain-enhancer of activated B cells (NF-kB) activation pathways normally triggered by pro-inflammatory cytokines, and contributing to tumour ‘tolerance’ [51]. Specific genetic events, such as alterations in the B-catenin/WNT pathway and loss of PTEN, may also impair intra-tumoral infiltration of functional antigen presenting cells, thereby diminishing the anti-tumour immune response [49]. Similarly, additional inhibitory proteins upregulated on the tumour cell surface, such as integrin associated protein (CD47), may affect efficacy of phagocytic cells and have been described to promote tumour progression and metastasis in MTC [52]. Ultimately, for a sustained anti-tumour immune response, activation and clonal expansion of key effector cells must be initiated and iteratively expanded [41], and if the TME disrupts this process through the balance of cytokines and chemokines, the cancer is able to escape immune control and progress to become clinically significant [42].

### 8.2. Immune Suppression through Surface Receptor Co-Stimulatory Inhibition

In addition to the cytokine milieu of the TME, cell-surface receptors play an important role in modulation of lymphocyte activation, through co-stimulatory and co-inhibitory ligand pairs. There is accumulating evidence that immune co-inhibitory receptors (CIRs) and their respective ligands on peri-tumoral lymphocytes interact within the TME, thereby facilitating immune evasion and escape [53]. Cytotoxic T-lymphocyte-associated protein 4 (CTLA-4) and programmed death receptor 1 (PD-1) have been demonstrated to be key regulators of T-cell anti-tumour immune response and are effective targets for immunotherapeutic agents [54,55].

#### 8.2.1. Cytotoxic T-Lymphocyte-Associated Protein 4 (CTLA-4)

CTLA-4 binds to CD80 and CD86 and is a co-stimulatory signal involved in T-cell activation and survival. Expressed on the T cell surface, CTLA-4 outcompetes CD28 for ligand binding and therefore inhibits T-cell activation [56]. However, tumour cells may adapt to express CTLA-4, modulating the anti-tumour immune response [57]. In MTC, it has been suggested that tumour-associated CTLA-4 expression is exclusive to sporadic cases [58] and may contribute to the stage-adjusted worse prognosis seen when comparing sporadic and hereditary disease [59].

#### 8.2.2. PD-1

The programmed death receptor (PD-1) [60] is expressed on activated T cells, B cells, monocytes, NK cells and DCs. Through the binding of its ligand PD-L1, it acts to inhibit the cytotoxic T cell response [61]. Tumours may harness this immunosuppressive mechanism through expression of PD-L1, adversely affecting prognosis in several cancers [62], including papillary thyroid cancer [63]. In MTC, these inhibitory pathways have not been extensively explored and their contribution to oncogenesis is unclear. Two small cohort studies have demonstrated very low PD-L1 expression on primary MTC tumours [30,64]. In a larger series of 201 consecutive primary MTCs, PD-L1 staining was observed in 14% of cases, with expression correlating with advanced TNM stage and prognosis [65].

In addition to the upregulation of PD-L1 on the surface of tumour cells, cancer cell-intrinsic expression of both PD-L1 and PD-1 may result in modulation of the PD-1/PD-L1 axis, with downstream activation of the mTOR signalling pathway [66]. In MTC, PD-1/PD-L1 co-expression may occur in up to 50% of PD-1 positive tumours [58,67], and correlate with advanced disease stage [67]. However, the clinical relevance of these findings remains uncertain.

The PD1/PD-L1 pathway can be disrupted by PD-1 inhibitors, such as pembrolizumab and nivolumab, which enhance tumour recognition by cytotoxic T cells [68,69], and have been shown to induce durable anti-tumour response for a variety of tumours, many of which were not considered to be particularly susceptible to immunotherapy [70,71]. However, predicting which patients will respond to therapy is challenging [72], and overall response rates to PD-1 inhibitors remain low (~20–25% of treated patients) [73]. In a small phase II study that included 7 patients with MTC, no pathological responses were seen with combination anti-PD1 and anti-CTLA4 treatment (nivolumab and ipilimumab), whereas in anaplastic thyroid cancer a partial response was seen in 3/10 of patients, including two with a complete response [74]. These findings require validation in larger cohorts; however, they suggest anti-PD1 immunotherapy has limited efficacy in MTC. This lack of efficacy may be affected by the low tumour mutational burden (TMB) seen in MTC and expression of additional regulatory molecules, such as polio virus receptor (CD155). Expressed in a variety of cancers including metastatic MTC, it interacts with CD8+ T cells to promote to immune evasion [30].

## 9. Emerging Co-Inhibitory Receptors

The T-cell immunoglobulin and mucin-domain containing-3 (TIM-3) has recently been identified as a potential therapeutic target [75]. When expressed on T-cells, TIM-3 is an indicator of T cell exhaustion in both chronic viral infections and malignancy [76,77], and its overexpression on tumour cells has been described in several solid tumours, including lung, gastric, colon, hepatocellular and urological malignancies [78], with increased levels correlating with poor survival [78,79]. Expression of TIM-3 has been described in 48% of primary MTC tumours and was associated with extensive locoregional metastasis, advanced stage and disease recurrence [58]. However, TIM-3 was expressed solely on tumour cells and not on tumour-infiltrating lymphocytes (TILs). Corroborating this finding, an analysis of TILs in MTC tumours demonstrated sparse TIM-3 expression [30]. The exact mechanism of MTC tumour intrinsic TIM-3 remains poorly understood but is thought to involve the nuclear-factor kB (NF-kB) pathway [80]. NF- kB encompasses a family of transcription factors involved in the regulation of cytokines and their receptors, as well as cell adhesion molecules [81], and may play a role in carcinogenesis through inhibition of apoptosis and promotion of cell cycle progression [80].

CD276 (B7 Homolog 3, B7-H3) is a recently described immune checkpoint molecule, that suppresses T cell activation and proliferation, and is overexpressed in a variety of malignancies including MTC [82]. It has been shown to promote tumour cell immune evasion and T cell inhibition, by altering secretion of pro-inflammatory cytokines [7]. In the context of MTC, CD276 was found to be expressed three-fold higher in tumour tissue, although correlation with histopathological factors and prognosis was equivocal [7,82].

### Tumour-Infiltrating Lymphocytes (TILs)

The presence of TILs is an indicator of anti-tumour immune activation and is associated with improved prognosis in several malignancies [83,84]. The International TILs Working Group (ITWG) published a standardised approach for assessment of TILs in breast cancer [85], which has since been applied to several other solid malignancies [83,84]. In brief, the ITWG approach reports TILs as a percentage of the surface area of the stromal component within the borders of invasive tumour, and excludes TILs outside this area, as well as in zones affected by crush artifact or necrosis from biopsy sites [85]. Using a standardised methodology for assessment of TILs is particular important in thyroid cancer, as T cell infiltration into the thyroid gland is a common histopathological finding, for example, in chronic lymphocytic (Hashimoto’s) thyroiditis [86]. In differentiated thyroid cancer, lymphocytic infiltration has been reported to correlate with improved survival [87] and lower rates of extrathyroidal extension [88], suggesting the immune response may be suppressing tumour growth. In MTC, data are limited. Scopsi et al. found lymphocytic infiltrate to be associated with a favourable prognosis; however, they commented that no true TILs were identified [89]. French et al. demonstrated that background lymphocytic infiltrate had no correlation with disease stage or prognosis, whereas true TILs were associated with advanced disease stage, locally invasive tumours and lymph node metastases [90]. Pozdeyev et al. reported organised immune cell infiltration (predominantly, CD8 T cells) in 49% of primary and 90% of metastatic MTC tumours [30]; however, this was not assessed using the ITWG approach, and the prognostic correlation requires further research.

The phenotypic subtype of TILs also influences the degree to which they recognise tumour antigens [91] and the response to immunotherapy. For example, not all TILs display signs of clonal expansion to tumour antigens and rather can be considered ‘bystander’ T cells (CD 39– CD8+ cells). A predominance of bystander cells may be associated with poor response to immunotherapy [92]. Furthermore, a high proportion of infiltrating regulatory T cells (Tregs—CD3 + CD4 + CD25 + FoxP3+), and a low CD8:Treg ratio have been demonstrated to correlate with tumour size and lymph node metastases in papillary thyroid cancer [90]. Hence, the absolute number of TILs alone may not necessarily imply an immunologically ‘hot’ [93] tumour or predict a favourable response to immunotherapy.

Tregs develop in the presence of TGF-β and temper the immune response to allow tolerance of self-antigens [94]. Within TME, they can impair immune clearance of tumour cells through secretion of immunosuppressive cytokines and expression of co-inhibitory cell surface molecules (e.g., CTLA-4) and have been demonstrated to be associated with tumour progression and reduced survival in several cancers [95]. They have also been shown to interfere with effector lymphocyte ionised calcium uptake, which disrupts the NF-kB signalling pathway required for T cell activation [96]. However, there are conflicting data in the literature regarding prognostic significance of Tregs; their role in the TME may differ according to tumour type, with improved prognosis reported with Treg infiltration in the setting of colorectal cancer [97] and squamous cell carcinoma of the head and neck [95]. This may be due to general immune infiltration by multiple T cell subsets, and some authors suggest that the CD8:Treg ratio may be a better indicator of balance between immune tolerance and activation within the TME [98]. Interestingly, Salama et al. noted that although infiltration of Tregs in the TME was associated with an improved prognosis in colorectal cancer, high Treg density in adjacent normal mucosa was associated with a worse prognosis [97]. In MTC, limited data suggest that Tregs in the TME have a negative prognostic impact [99]; however, this hypothesis requires further empiric research.

## 10. Efficacy of Immunotherapy

Utilisation of immune checkpoint inhibitors (ICIs) for advanced disease has improved survival in several solid malignancies, most notably melanoma [100], non-small cell lung cancer [101], renal-cell carcinoma [102] and colorectal cancers with mismatch repair deficiency [103]. However, ICI therapy is not effective in all patients. Tumour mutational burden (TMB), the number of somatic mutations seen per megabase of tumour genome, has been demonstrated to be a predictive biomarker for response to ICI therapy, with increased TMB (>10 mutations/Mb) associated with greater response rates [104,105]. In tumours with <10 mutations/Mb, the objective response rate to ICI therapy is only 6% [104]. The underlying mechanism of ICI therapy efficacy with increased TMB likely relates to the higher proportion tumour-specific neoantigens that develop as a consequence of increased TMB [106], which can then be displayed on major histocompatibility complex (MHC) molecules of the tumour cell and recognised by T cells [107]. In comparison to most other solid tumours, MTC is associated with a low TMB, with the majority of tumours harbouring <1 mutation/Mb [108]. Most MTC tumours are driven by *RET* mutations, with few other mutated somatic genes [109], suggesting the MTC tumorigenesis pathway does not depend on the same degree of accumulation of genetic driver events required in other solid tumours, such as the adenoma–carcinoma sequence of colorectal cancer [110], explaining the low observed TMB. Therefore, it is unlikely that MTC patients will benefit from ICI therapy.

Pathogenic variants in genes encoding the antigen processing and presentation apparatus also affect the response to immunotherapy. Regulation of MHC proteins on the cell surface facilitates antigen presentation and determines ‘visibility’ of the cell to the immune system. Epigenetic modulation of MHC-I expression is required in utero for the foetus to avoid maternal immune attack of paternal MHC-I alleles, and this evolutionarily-preserved mechanism can also be exploited by cancer cells, particularly neuroendocrine tumours, to evade immune surveillance [111], with downregulation of MHC class I surface proteins and loss of B2-microglobulin. The clinical relevance of changes in MHC protein expression in MTC requires further evaluation, with only limited data published to date [30,112].

## 11. Current Systemic Treatment Options in MTC

### 11.1. Targeted Therapy with Pathway Inhibition

**Figure 2:**
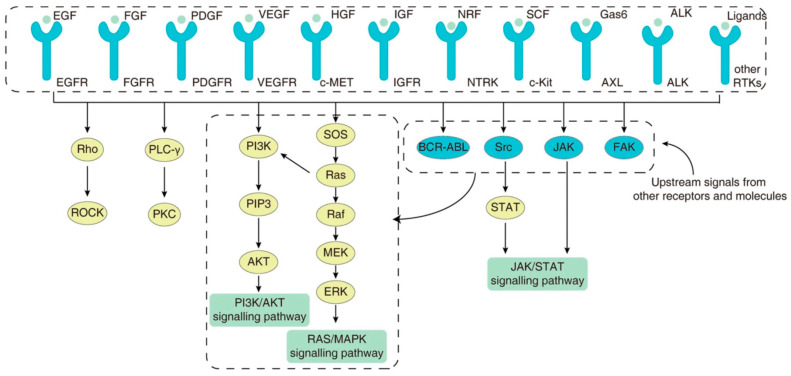
The variety of surface tyrosine kinase receptors that play an important role in the maintenance of cellular homeostasis, through regulation of intracellular signalling pathways. The final effector pathways can be activated by several surface receptors, as well as by other intracellular activation pathways, highlighting the inherent difficulty in achieving sustained therapeutic efficacy through the blockade of a particular tyrosine kinase receptor. Adapted from [118]. Published under a Creative Commons Attribution (CC BY) License.

Although MTC is associated with a low TMB, the majority of driver events are clinically actionable with currently approved pathway inhibition therapies [113,114]. TKIs are homologs of adenosine triphosphate (ATP) and competitively occupy the ATP binding sites of tyrosine kinase receptors, thus inhibiting activation of associated signalling pathways [115]. The RET TKR shares similarities with other tyrosine kinases and therefore can be targeted by both multitarget tyrosine kinase inhibitors (MKIs) and selective tyrosine kinase inhibitors. MKIs, including vandetanib and cabozantinib, act on several TKRs, including the VEGF receptor, platelet derived growth factor (PDGF) receptor, hepatocyte growth factor (c-MET) receptor, epidermal growth factor receptor (EGFR) and the RET TKR; although, the dominant contribution to therapeutic efficacy comes from VEGF receptor inhibition (Figure 2). Given the broad range of receptor targets, MKIs are also associated with significant off-target toxicities, particularly affecting the gastrointestinal tract and liver. Among patients treated with vandetanib and cabozantinib, 35% and 79% required a dose reduction, and 12% and 16% required permanent discontinuation, respectively [116,117].

The need for better response rates and tolerability led to the development of selective RET inhibitors; selpercatinib [119] and pralsetinib [120]. Clinical trials have demonstrated lower rates of treatment discontinuation (2%) and reduced the severity of adverse effects associated with selpercatinib treatment compared to MKIs [121], with a significantly better objective response rate of 73% [119]. A recent phase 3 trial of selpercatinib in advanced RET-mutant MTC demonstrated a 12-month progression-free survival rate of 86.8% in comparison to 65.7% for the MKI control group, with 38.8% of patients requiring dose reduction due to toxicities vs. 77.3% in the MKI group [122]. The median progression-free survival had not been reached at the time of publication, and additional follow-up is required to define its ultimate durability.

### 11.2. Escape of Pathway Inhibition

Efficacy of TKIs may be limited by both ‘on-target’ and ‘off-target’ resistance mechanisms (Figure 2). On-target resistance refers to mutations that affect binding in the target kinase domain, whereas off-target resistance occurs through upregulation of bypass pathways [121,123].

## 12. On-Target Resistance

A large proportion of on-target mutations involve structural alteration of the receptor ATP-binding pocket (steric inhibition), which renders the drug inactive [124]. This may be an intrinsic or acquired mutation. For example, resistance to vandetanib occurs in MTCs with RETp.Val804Met ‘gatekeeper’ variants [121], in which a conformational change to spatial arrangement of the binding site prevents vandetanib from binding. In this situation, the amino acid substitution results in a greater hydrophobic force that impedes binding of the TKI to the receptor. Similarly, tumour-specific mutations in the target tyrosine-kinase receptor may confer intrinsic resistance to TKI treatment, such as EGFR mutations in patients with lung adenocarcinoma [125] and PDGFR in gastrointestinal stromal tumours [126].

In addition to gatekeeper variants, specific changes in exposed kinase residues of the receptor binding region (‘solvent front mutations’) may confer resistance. For example, the RET solvent front variant G810A results in the addition of a methyl group, which creates hydrophobic disruption of vandetanib binding, but still allows binging of nintedanib due to presence of a corresponding methyl group absent in vandetanib. Hence, RET G810A confers resistance to vandetanib, but not nintedanib [127]. Similarly, RET L881V-driven tumours are resistant to lenvatinib, cabozantinib and vandetanib due to absence of a phenyl ring required for binding [127], and presence of this phenyl ring on RET L730V confers resistance to nintedanib, due to a hydrophobic interaction [118]. Such solvent front mutations may be acquired during treatment because of selection pressure. For example, following an initial dramatic response to selpercatinib, emergence of RET G810R, G810S and G810C solvent front mutations result in the development of on-target resistance [128]. Furthermore, RET V804L/M and G810S mutations may confer pan-TKI resistance to the MKIs and RET-specific treatments [124]. Bypassing these mechanisms of resistance through the design of structurally different RET inhibitors is an ongoing area of research focus [129].

The binding affinity of TKIs may also be moderated by TKR mutations and hence also contribute to treatment resistance. For example, the RETS904F mutation has been demonstrated to increase the autophosphorylation of the mutant RET kinase, as well as the its ATP binding affinity, both of which reduce the efficacy of MKI therapy [130].

## 13. Off-Target Resistance

**Figure 3:**
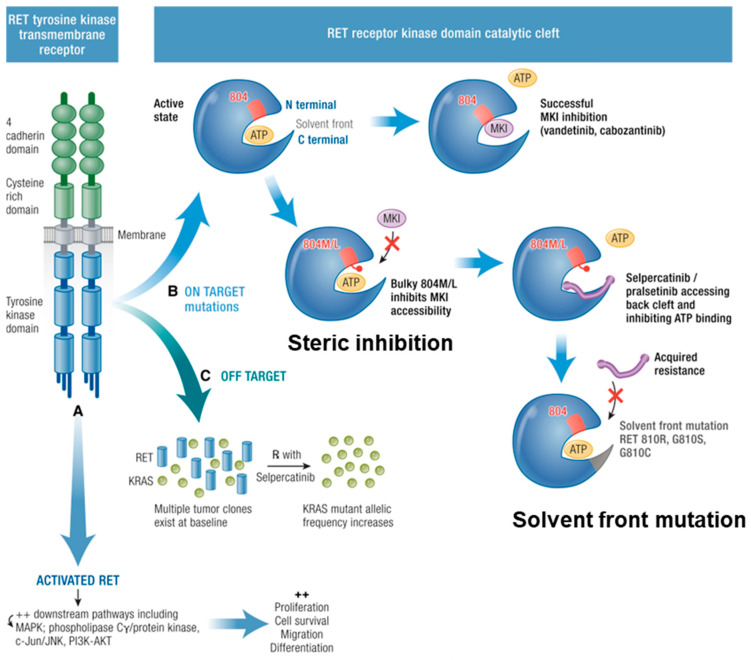
Mechanisms of MKI resistance. RET tyrosine kinase transmembrane receptor has constitutive activation due to RET point mutations leading to downstream pathway activation. (A) Resistance mechanisms are described. (B) On target: the RET receptor kinase domain catalytic cleft is activated when ATP causes phosphorylation. MKIs (vandetanib, cabozantinib) can hinder the ATP binding when there is no V804M/L mutation with bulky hydrophobic side chains: steric inhibition. Acquired solvent front mutations RET G810R, G810S, G810C hinder this binding rendering selpercatinib ineffective. (C) Off Target mutations are shown with multiple tumour clones existing at baseline, reduction in RET, and subsequent increase in KRAS (or MET) allelic frequency. Adapted from [121]. Published under a Creative Commons Attribution (CC BY) License.

Off-target resistance can develop when proliferative pathways are activated by alternative mechanisms, such as MET [131] and KRAS that bypass the targeted kinase [121,130] (Figure 3). Because several receptor tyrosine kinases can activate the same downstream pathways (PI3K/AKT and RAS/MAPK), the therapeutic effect of blocking a particular tyrosine kinase receptor is reduced when tumour cells driver activation of the downstream pathways through alternative TKRs [132,133].

The selection pressure created by prolonged treatment allows selection of resistant clones, in which mutations in the TKR may result in constituent activation, limiting the efficacy of TKIs. In addition, resistance may develop through cytokine and chemokine modulation of the TME [134]. For example, the presence of inflammatory cytokines such as tumour necrosis factor-alpha may modulate the downstream pathways of the EGFR and hence reduce the efficacy of EGFR-targeted TKIs [135]. Similarly, chemokine signalling mediated through chemokine receptor 2 activates the downstream PI3K/AKT pathway which has been demonstrated to impact the efficacy of TKI therapy [136]. Furthermore, immunosuppressive M2 macrophages may reduce the efficacy of TKI therapy through the production of chemokines which can modulate both surface receptors such as MET [137] and downstream intracellular pathways [134].

Additional mechanisms of off-target resistance include the modification of the metabolic cellular pathways [138] and epigenetic modification, including DNA methylation [139], histone modifications [140] and mRNA modification [141]. Lastly, adaptive mechanisms to alter drug metabolism and limit intracellular drug concentration, including lysosomal sequestration and drug efflux, may also contribute to development of off-target resistance [118].

## 14. Conclusions and Future Directions

In this review, we highlight that MTC is consistently driven by a small number of specific pathogenic variants, beyond which few additional genetic events are required for tumorigenesis. This homogeneity of driver events explains the exceedingly low tumour mutational burden seen in MTC, in contrast to other cancers. However, as a result, there is a correspondingly low level of tumour-associated neoantigens presented to the host immune system. This reduces tumour visibility and the vigour of the anti-tumour immune response. In addition, it suggests the efficacy of immunotherapy in MTC is likely to be poor, acknowledging this inference is largely based on the extrapolation of data from other tumour types. Specific to MTC, the immune microenvironment has not been extensively described, with conflicting data published to date. Correlation of the cytokine and immune cell profile of the TME with the underlying molecular subtype, clinicopathological factors and prognosis, as well as description of changes that occur in the TME with TKI therapy remain important areas for future research.

The dominance of specific *RET* pathogenic variants in MTC tumorigenesis rationalises the observed superior efficacy of the targeted RET TKIs in comparison to MKIs. Therapeutic durability of RET-specific pathway inhibitors is also superior to that of the MKIs; however, the development of resistance to pathway inhibition remains an inherent limitation of TKI treatment. Resistance may develop through the selection pressure TKI treatment creates, promoting survival of resistant tumour cell clones that can escape pathway inhibition through binding-site mutations, activation of alternate pathways, and modulation of the cellular and cytokine milieu of the TME. The optimal therapeutic strategies to delay the emergency of resistance and the approach to management once resistance occurs remain important areas for future research.

## Data Availability

Not applicable.
